# Wearable Sensors in Sports for Persons with Disability: A Systematic Review

**DOI:** 10.3390/s21051858

**Published:** 2021-03-07

**Authors:** Lorenzo Rum, Oscar Sten, Eleonora Vendrame, Valeria Belluscio, Valentina Camomilla, Giuseppe Vannozzi, Luigi Truppa, Marco Notarantonio, Tommaso Sciarra, Aldo Lazich, Andrea Mannini, Elena Bergamini

**Affiliations:** 1 Interuniversity Centre of Bioengineering of the Human Neuromusculoskeletal System, Department of Movement, Human and Health Sciences, University of Rome “Foro Italico”, Piazza L. De Bosis 6, 00135 Rome, Italy; lorenzo.rum@uniroma4.it (L.R.); valeria.belluscio@gmail.com (V.B.); valentina.camomilla@uniroma4.it (V.C.); elena.bergamini@uniroma4.it (E.B.); 2 BioRobotics Institute, Scuola Superiore Sant’Anna, 56025 Pisa, Italy; o.sten@santannapisa.it (O.S.); eleonora.vendrame@santannapisa.it (E.V.); l.truppa@santannapisa.it (L.T.); a.mannini@santannapisa.it (A.M.); 3 Joint Veteran Center, Scientific Department, Army Medical Center, 00184 Rome, Italy; marco.notarantonio@am.difesa.it (M.N.); tommaso.sciarra@esercito.difesa.it (T.S.); casezricveterani@policlin.esercito.difesa.it (A.L.); 4 IRCCS Fondazione Don Carlo Gnocchi, 50143 Firenze, Italy

**Keywords:** sport technology, athletes, biomechanics, inertial sensors, electromyography, paralympic

## Abstract

The interest and competitiveness in sports for persons with disabilities has increased significantly in the recent years, creating a demand for technological tools supporting practice. Wearable sensors offer non-invasive, portable and overall convenient ways to monitor sports practice. This systematic review aims at providing current evidence on the application of wearable sensors in sports for persons with disability. A search for articles published in English before May 2020 was performed on Scopus, Web-Of-Science, PubMed and EBSCO databases, searching titles, abstracts and keywords with a search string involving terms regarding wearable sensors, sports and disability. After full paper screening, 39 studies were included. Inertial and EMG sensors were the most commonly adopted wearable technologies, while wheelchair sports were the most investigated. Four main target applications of wearable sensors relevant to sports for people with disability were identified and discussed: athlete classification, injury prevention, performance characterization for training optimization and equipment customization. The collected evidence provides an overview on the application of wearable sensors in sports for persons with disability, providing useful indication for researchers, coaches and trainers. Several gaps in the different target applications are highlighted altogether with recommendation on future directions.

## 1. Introduction

### 1.1. Background

Over the last few years, the interest in sports for persons with disability has grown at an impressive rate. The Paralympic winter games of 2018 in PyeongChang hosted 343 thousand spectators, which was twice the attendance at Turin 2006 games, and had a cumulative international TV audience of 2.02 billion views [1]. Simultaneously, the participation in sports by a growing number of persons with disabilities has been observed, with evidence showing the positive impact of sport on quality of life, physical health and psycho-social wellbeing in this population [2,3,4]. Given the large variety of disabilities and how they specifically affect and influence the sports practice, advances in research and technology play a key role in providing tools for a safe, inclusive and effective participation in sport.

### 1.2. Wearable Technologies in Sport

To date, technology has been used to improve and support the athlete’s training and development in both elite and amateur sports for non-disabled and people with disabilities [5,6,7,8]. Many technologies are currently available to monitor sport performance and one of the most represented tools is motion capture. Stereophotogrammetric systems are widely regarded as the gold standard for motion capture, as they are the most accurate technique to track the kinematics of human movement [9]. However, this technology can only be used in a small area of observation and requires time and skill for the calibration procedures, thereby being mostly adopted in laboratories than in outdoor or in-field environments [9]. Video analysis and radio frequency tracking systems are also frequently used for movement analysis in sports, although less accurate and informative than stereophotogrammetric systems [10,11]. In the last decades, an increase in the application of wearable technologies in the sport field has been observed as they can be used with less restrictions compared to the above-mentioned technological tools [5,12,13]. These technologies are adopted to measure different components of an athlete’s movement as well as to explore the relation between the athlete’s body and the sport equipment. Several relevant kinematic and kinetic parameters can be estimated with inertial sensors [5,12,13], while other characteristics of movement in space are measured by pedometers [14], GPS [11,14] and position data loggers [11]. The force output that is exerted through the athlete motion during the sport gesture can also be measured by different types of force sensors [11,13]. Further wearable sensors, such as heart rate sensors, wireless electromyography (EMG) devices and portable metabolimeters, allow to measure and track physiological parameters in many different conditions [13,15,16]. From the results of two recent reviews [12,13], inertial and EMG sensors appeared to be the most widely used wearable sensors in sport biomechanics.

The inertial sensors used in sport applications are typically based on microelectromechanical system (MEMS) technology, which allows to realize small, light-weight and relatively affordable wearable devices. These MEMS sensors typically refer to accelerometers and gyroscopes with one, two or three sensing axes that are often combined into an inertial measurement unit (IMU). Often, a 3D magnetometer is also included; in this case, the term magneto-inertial measurement unit (MIMU) is commonly used [5]. An accelerometer measures the acceleration along its sensitive axis, including the gravitational acceleration. It can measure linear acceleration in a given direction and, when in quasi-static conditions, assess sensor inclination with respect to gravity. In addition, when a person moves, it allows to measure different acceleration patterns depending on the movement. Therefore, analyzing features of the accelerometer signal can aid, for example, in identifying movement type, analyzing its characteristics or detecting pathological alteration of the movement pattern [17,18,19]. Theoretically, once the contribution of gravity on each sensor axis is known and removed, an accelerometer could be used to track position through double integration of the inertial acceleration but, in practice, the presence of noise leads to unbounded integration drift [9]. A gyroscope measures the angular velocity around its sensitive axis. Three-dimensional orientation can be obtained by numerically integrating this signal within the framework of the kinematic differential equations that relate the time derivatives of the orientation parameters to angular velocity. However, the accuracy of this integration is hindered by errors due to integration drift. Moreover, the initial conditions of the integration process must be determined. To this aim, magnetometers can be used to obtain complementary information to the accelerometer for the definition of a 3D inertial system of reference. Magnetometers measure the Earth’s magnetic field vector components, whose projection on the horizontal plane is used to define an axis orthogonal to gravity. Therefore, it allows the estimation of the orientation in the horizontal plane which cannot be obtained using accelerometers, though it is strongly affected by magnetic disturbances. Despite all elements in a MIMU having limitations, the nine-dimensional MIMU signals can be used to accurately estimate the sensor’s orientation in a global frame defined using the gravity and magnetic North directions [19]. This is possible thanks to the redundancy of information achievable by merging accelerometers, gyroscopes and magnetometers, using ad hoc sensor fusion techniques, such as complementary or non-linear Kalman filtering [20].

Surface EMG sensors register electrical muscle activity at the skin site over the muscle belly, with bipolar setups being the most commonly used [21,22]. The summation of consecutive action potentials is registered during an observed motor task and post-processed to remove noise or to normalize the signal for inter- and intra-subject comparison [23,24]. EMG signal provides information to quantify muscle effort, through signal rectification and integration or the computation of peak amplitude, and to identify specific muscle activation patterns and synergies, which are defined by temporal events (i.e., onset and offset of muscle activation) [25,26]. In sport applications, EMG analysis is commonly performed to assess muscle activation amplitude or to detect EMG activity onset and offset; in further cases, frequency analysis allows the estimation of muscle fatigue [13]. Nowadays, the advent of commercially available, wearable and portable wireless EMG systems favors the study of how the movement is executed and controlled by the central nervous system. In fact, surface EMG sensors are also embedded into athletic garments for their use in indoor and outdoor environments [15].

### 1.3. Applications of Wearable Technologies in Sport for People with Disabilities

Given their ecological and versatile properties, wearable sensors can provide objective measurement methods that can be applied in real sport-life situation and finely fit within several purposes. Aside from those general to all athletes population, the following aspects are specific to athletes with disabilities: athlete classification, sport equipment customization, and monitoring the athlete’s technique to prevent injury while designing successful training protocols.

One of the greatest challenges in the use of technological tools to assess sport performance of people with disabilities is that the disability rarely affects two individuals in the same manner, thereby introducing larger inter-subject variability with respect to able-bodied individuals [10]. In Paralympics and other sport competitions, athletes with disabilities compete in different classes or categories in accordance with the principle that “classification is undertaken to ensure that the impact of impairment is minimized and sporting excellence determines which athlete or team is ultimately victorious” [27]. In 2009, the International Paralympic Committee (IPC) Position Stand on Classification in Paralympic sport [28] promoted the development of such classification systems to increase participation in sport among people with disabilities by minimizing the impact of impairment on the outcome of competition. To move forward with respect to assigning the class/category in which an athlete competes based on the subjective evaluation by experts, the development of evidence-based classification based on technological tools have become fundamental. The IPC’s handbook states that the impairment type and severity should be considered when classifying athletes, with 10 types of impairment being currently recognized in the Paralympic classification. In this regard, research should develop objective and reliable measures of both athlete’s severity of impairment and related functional limitation, also investigating the association between the two in a large representative sample [28]. How much a given impairment with a given severity affects an athlete ability to perform a given sport-related task is thus a question to be answered through large scale studies, and wearable systems represent a feasible and practical solution to accomplish this purpose.

Furthermore, the large variety of impairments in athletes with disabilities also influences sport equipment design. In modern competitive sport, equipment plays a central role as the technological developments in manufacturing have provided both tools and materials to improve its ergonomics and performance-enhancing properties. While this is true for non-disabled athletes, it becomes even more relevant in athletes with disabilities, who often use assistive devices in the everyday life and require individual-specific adjustments to their equipment during the sport practice [10]. From wheelchair sports to winter Paralympic sports, the need to assess performance outcomes in relation to both equipment and athlete-equipment interface, particularly in condition of real sport practice, has become fundamental for improving sport equipment design [6,7,8,29].

Finally, monitoring the athlete’s technique directly in field through wearables is beneficial to all athletes to prevent injury while designing successful training protocols. Specific to athletes with disabilities, performance can be assessed in consideration of their impairment and adjusted on a quantitative rather than qualitative base. As stated by Curran et al. [10], the kinematic analysis of performance in sport for people with disability is the most important element for evidence-based training. Therefore, technical solutions that provide quantitative information about the athlete’s technique are fundamental to reinforce correct movement execution and to avoid injuries [11]. This application builds upon general evidence on the use of wearable sensors to monitor performance for training optimization [5,12] or modifiable risk factors with the aim of preventing injury [30,31].

### 1.4. The Aim

Previous reviews on the applications of wearable technology in sports did not specifically focus on athletes with disabilities, providing more general indications on the topic [5,12,13,32,33]. Literature does however highlight wearable sensors advantages and potential to support athletes with disabilities at all athletic levels and in different application fields. Therefore, the aim of this review was to provide information to future researchers, athletes and trainers to support evidence-based practice by exploring literature regarding the use of wearable sensors in sport for people with disabilities. Within this framework, we assessed which sports and motor tasks have been studied, which type of wearable sensors have been used for extracting which parameters and the available evidence on whether the four aforementioned applications (e.g., athlete classification, sports equipment customization, injury prevention and performance characterization for training optimization) were implemented using wearable sensors.

## 2. Methods and Material

### 2.1. Search Strategy

The systematic search was carried out from the Scopus, Web of Science, EBSCO and PubMed databases until May 2020. The keywords within the search strategy were grouped into three categories: wearable sensors, sport and disability (for more details on the search strings, please refer to the Appendix A). The wearable sensors category included the terms for the different type of sensors, such as accelerometer, gyroscope, IMU, electromyography, force transducer, pressure sensor, and devices for heart rate and oxygen consumption monitoring. The sport category included the taxonomy of sport activities related to the 28 Paralympic sports sanctioned by the International Paralympic Committee (IPC, https://www.paralympic.org/sports (accessed on 14 May 2020)) and other forms of sport that can be practiced by people with disability. The disability category included general terms for disability and impairment.

### 2.2. Eligibility Criteria

Criteria for inclusion/exclusion are summarized in Table 1. Only articles published in English were considered for inclusion. Articles were excluded if they were a review or short conference/congress abstracts, while case-report studies were included. Since the aim of the review was to collect current evidence on kinematic, kinetic and physiological parameters obtained through wearable sensors in sport for people with disability, only studies using sensors which are portable or mounted either on the body or equipment were included. To gather information relevant to the context of sport practice, papers investigating motor tasks other than sport-related movements, i.e., daily physical activity, were excluded. Studies were included only if they involved human participants: (a) with disability and (b) non-disabled performing a sport-related activity typically performed by athletes with disabilities (adaptive sport tasks, e.g., handcycling). To reduce population heterogeneity related to different types of disability, papers that involved people with cognitive disability only were excluded.

### 2.3. Review Process

The retrieved articles were imported into the Rayyan online software (http://rayyan.qcri.org (accessed on 14 May 2020) [34]) and duplicates from the multiple database search were removed. The review process of title and abstract was performed by two independent reviewers (L.R. and O.S.) according to the inclusion and exclusion criteria. Both reviewer’s results were compared through discussion and any conflicts were discussed and resolved by the consensus of other authors (E.B., A.M., V.C., G.V., V.B., L.T.). Full text papers were then retrieved and further evaluated for inclusion according to the eligibility criteria.

### 2.4. Quality Assessment

The quality assessment of the included papers was performed by two reviewers independently (L.R. and O.S.) adopting the 14-item checklist proposed by Kmet and colleagues [35]. Each item of the scale had three levels of scoring (yes = 2, partial = 1, and no = 0) and any conflict between reviewer’s opinion was resolved through discussion and consensus. A final quality score was obtained for each paper by dividing the sum of all item scores by the highest possible score, with the score ranging from 0 (low quality) to 1 (high quality).

### 2.5. Data Extraction

The following details were extracted from the included articles: publication year and journal; investigated sport and sport-related movement; type of disability; aim; type of target application (athlete classification, injury prevention, training optimization or equipment customization); setting (in-field or laboratory); athlete level (elite or amateur); sample size and relevant details (number of participants with/without disability and grouping characteristics); type of sensors adopted; sensor setup (placement and data transmission); parameter extracted from specific sensors; data acquisition and processing (sampling frequency and filtering); main findings and final conclusions.

## 3. Results

**Figure 1:**
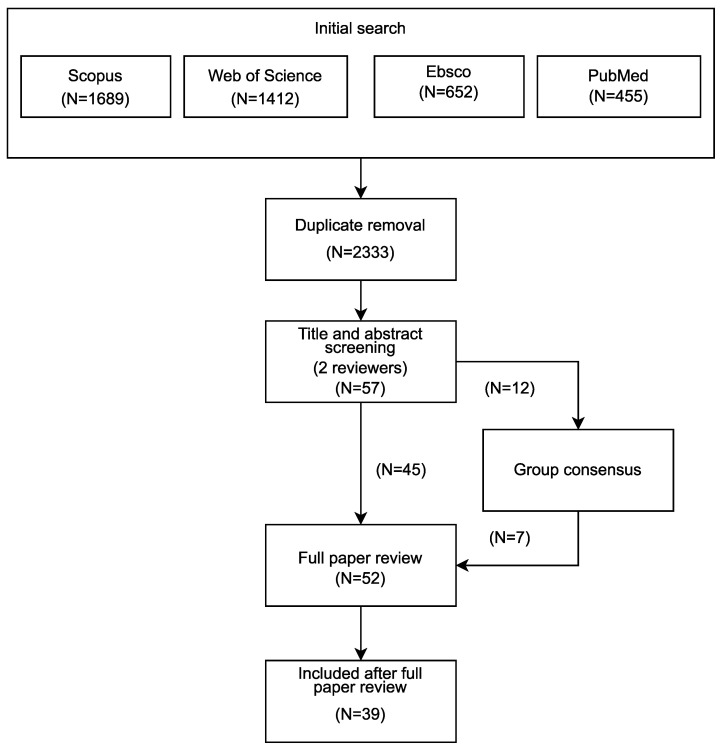
Flowchart of the screening process.

The multiple database search yielded a total of 4208 articles, reduced to 2333 articles after duplicate removal (Figure 1). Fifty-seven papers resulted from the screening of title and abstract, of which 45 papers were directly included after reviewers agreement. Conflicts for the remaining 12 papers were resolved through all authors consensus, with 5 papers being discarded. After full text review, a final number of 39 papers were included. The data extracted from each study are provided in detail in the Supplementary Material (Main Table).

As regards quality assessment results, 28 papers had a quality score between 0.81 and 1 [36,37,38,39,40,41,42,43,44,45,46,47,48,49,50,51,52,53,54,55,56,57,58,59,60,61,62,63], 10 papers were between 0.61 and 0.8 [64,65,66,67,68,69,70,71,72,73] and only one paper was below 0.6 [74]. Overall, the checklist items that reported the lower levels of scoring (e.g., partial or no score) were those concerning *(a)* the adequacy of sample size (16 papers), *(b)* the description/appropriateness of the strategy of subject/comparison group selection (10 papers).

### 3.1. Journal and Year of Publication

**Figure 2:**
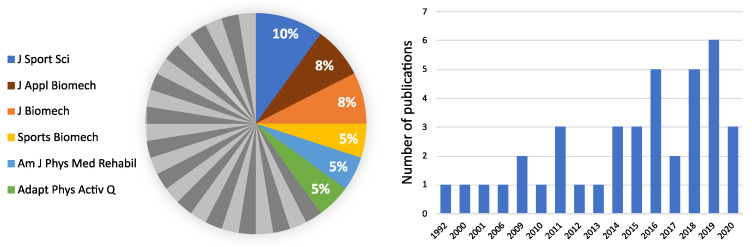
Distribution of included papers over journals (in %, **left** panel) and time (year of publication, **right** panel). Journals from which only one paper was retrieved are displayed in dark and light grey.

The research interest on the use of wearable sensors in sport for people with disability has been growing in recent years, with about 59% of the included papers being published in the last 5-year period (Figure 2). The included papers were mainly published in journals with a focus on sport science and biomechanical fields, followed by journals with interest in the biomedical area (Figure 2).

### 3.2. Sport, Motor Task and Setting

A large variety of sports were investigated, including team sports, cyclic sports and other sports (Table 2). The most common were wheelchair sports, with studies on wheelchair basketball [41,42,49,64,71,73], rugby [36,43,44,45,51,66], racing [40,47,53,54,67], tennis [50,63] and curling [48] covering almost 50% of the analyzed papers. The other half included cyclic sports (running [60,61,62,72], handcycling [57,58,59,70], swimming [37,38,39], cycling [52,65], rowing [46]) and other sports (weightlifting [69], boccia [55], cross-country sit-skiing [56,68] and downhill skiing [74]).

As regards considered motor tasks, Table 3 summarizes for each sport-related movement considered in the included papers, the wearable sensors used and the derived parameters. In the papers regarding wheelchair sports, different aspects of wheelchair propulsion were investigated, such as forward propulsion [64], complex maneuvers (i.e., turning and sprinting) [41] or motion tracking during game-play [36]. As regards the other sports, sport-specific movements (i.e., handcycling, cycling, running, rowing, bench pressing, throwing a ball in boccia, etc.) or their components (i.e., kicking in freestyle swimming) were analyzed. In some cases, the tested movement was not strictly related to the sport, but part of the assessment of athlete’s characteristics, such as vertical jumping in running athletes [61] or pushing/pulling force in cross-country sit-skiing [68] and wheelchair athletes [71].

The experimental setting was in the laboratory in 13 studies [40,46,47,52,53,54,55,57,58,59,68,70,71], while in 23 studies testing was performed in the field [36,37,38,39,41,42,43,44,45,49,50,51,60,61,62,63,64,65,66,69,72,73,74]. Both laboratory and in-field setting were observed in one study comparing cross-country sit-skiing performance in the laboratory and in a skiing tunnel [56], whereas information about the setting was not clearly retrievable from two studies [48,67].

### 3.3. Participants

The sample in the studies included in the systematic review involved participants with disability (27 papers), non-disabled participants (6 papers, [40,57,58,59,67,70]) or a combination of the two categories (6 papers, [46,52,60,61,62,71]). When participants with disability were involved, the type of disability was not always disclosed (e.g., disability classification score being reported for the entire sample or for each participant) or was heterogeneous within the sample, with different disabilities being represented. The most common disabilities were upper/lower limb dysfunction/deficiencies (9 papers), cerebral palsy (8 papers), spinal cord injury (8 papers) and upper/lower limb amputation (6 papers). Other types of disabilities were transient osteoporosis, multiple sclerosis, neuromuscular disorders and brain injuries.

Sample size was lower than 10 participants in 31% of the included papers, with seven of them being case-studies [40,45,48,51,68,70,74], 51% involved 11 to 20 participants and 18% had a sample size greater than 20 participants. Only one study had a sample size greater than 30 (52 participants) [36].

### 3.4. Sensor Types and Placements

**Figure 3:**
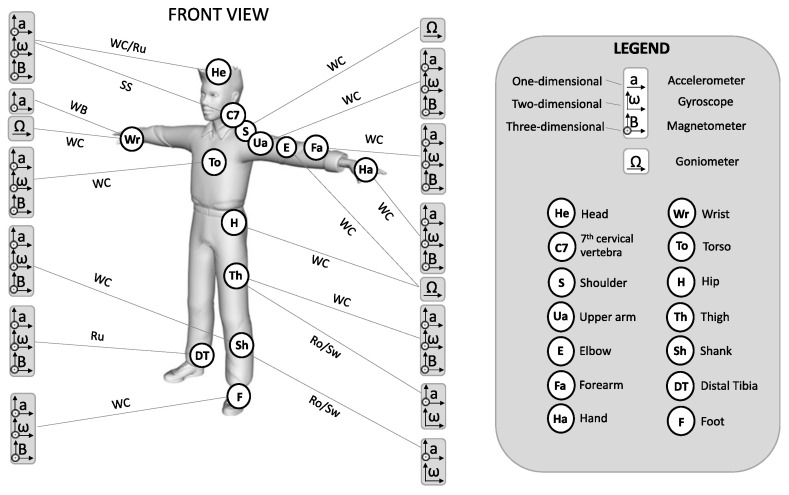
Configuration and positioning of inertial sensors on the athlete’s body. Coding for different body positions, sensor type and number of dimensions is displayed in the legend box. WC = Wheelchair curling [48]; WB = Wheelchair basketball [64]; Ru = Running [72]; SS = Sit-skiing [68]; Ro = Rowing [46]; Sw = Swimming [37,38,39].

**Figure 4:**
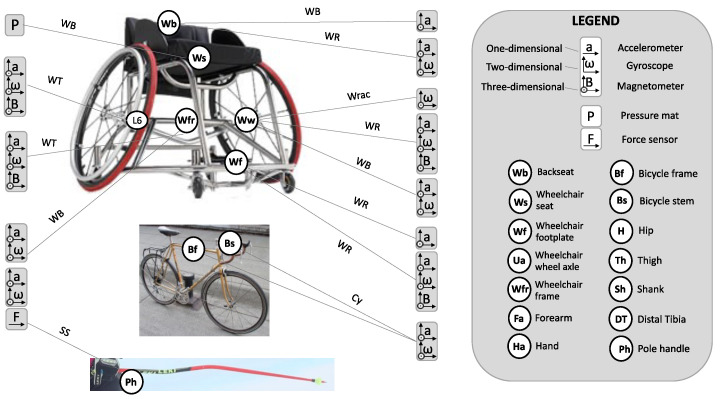
Configuration and positioning of wearable sensors mounted on sports equipment. WB = Wheelchair basketball [41,42,49,64,73]; WR = Wheelchair rugby [43,44,45,66]; WT = Wheelchair tennis [50,63]; Wrac = Wheelchair racing [40]; SS = Sit-skiing [56,68]; Cy = Cycling [65].

**Figure 5:**
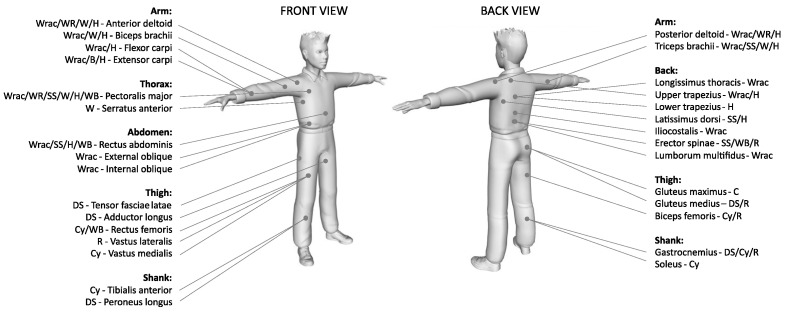
Positioning of EMG sensors with the specific muscle and related sport. Wrac = Wheelchair racing [47,53,54,67]; WR = Wheelchair rugby [51]; WB = Wheelchair basketball [71]; DS = Downhill skiing [74]; Cy = Cycling [52]; B = Boccia [55]; SS = Sit-skiing [56]; W = Paralympic weightlifting [69]; H = Handcycling [57,58,59,70]; R = Running [60,61,62].

The wearable sensors mainly adopted in the analyzed papers were inertial and EMG sensors, with the former being placed on the body of the participant or on the sport equipment. Sport-specific configuration and placement of inertial and EMG sensors are summarized in Figure 3, Figure 4 and Figure 5. Other types of wearable sensors and their placement were: force sensors placed on sports equipment [39,52,56,68,70,71], a pressure mat positioned at the body-equipment interface [73], digital goniometer [48] and heart rate sensors [49,61,62] placed on the participants’ body, and a GPS mounted on the sport equipment [63].

### 3.5. Parameters and Applications

Table 3 shows the parameters, specific to each motor task, extracted from different types of wearable sensors. The main applications for wearable sensors were athlete classification, injury prevention, performance characterization for training optimization, and sport equipment customization. Parameters and results obtained for each application area are presented in detail in the following subsections.

#### 3.5.1. Athlete Classification

Three studies explored athlete classification using wearable sensors in cross-country sit-skiing [68], wheelchair basketball [71], and boccia [55]. In the qualitative case-study by Rosso et al. [68], a custom-made device was used to assess the subject’s ability to control the trunk segment while generating force. A test was designed to classify cross-country sit-skiers, for whom the trunk segment plays an important role for propulsion generation and balance maintenance. During different testing conditions, trunk range of motion and angular velocity were measured with two inertial sensors mounted on the cervical vertebra and on the bottom of the device frame. Although preliminary, results from the tests with the custom-made device showed its potential for use in future studies and evidence-based athlete classification. In a study performed on participants both with and without disability to classify wheelchair basketball players, Rehm and colleagues [71] evaluated the athlete’s ability to produce pushing forces against a wall-mounted force gauge in various sitting positions, while measuring the EMG activity of trunk muscles. Similar to cross-country sit-skiing, the objective evaluation of trunk movement capacity and its contribution in wheelchair propulsion and balance maintenance is fundamental for evidence-based athlete classification in wheelchair-based sports. In this study, an EMG device was used to identify the neuromuscular strategies adopted to deal with the tasks. The researchers found a significant difference in force production with similar levels of EMG activity between participants with and without disability, although it was not clear to what extent the presented test could be able to stratify athletes based on the level of disability. In the study by Vaíllo et al. [55], neuro-mechanical features of the movement of boccia players were evaluated to explore key aspects for athlete classification. An EMG device was used to assess the electromechanical delay between the onset of finger extensor activation and ball release, elected as a good parameter for athlete classification. However, no significant difference between groups was found and the electromechanical delay was suggested not to have enough sensitivity for classification.

#### 3.5.2. Injury Prevention

Injury prevention was dealt with in three studies using different types of sensors directly in-field [51,72,73]. In their case-study, Barfield et al. [51] used an EMG device to measure shoulder muscles activity from an elite wheelchair rugby player during actual training sessions, with the intent of quantifying agonist-antagonist imbalances that could increase the risk of shoulder pain and injury. The results indicated that fatigue was equally present in both agonist and antagonist muscles related to wheelchair pushing, across and within training sessions. In another study, Shafizadeh and colleagues [72] used IMUs to investigate the capacity of impact shock absorption in athletes with neurological disabilities while sprinting with a RaceRunning bike in an indoor athletic track. The attenuation of acceleration peak amplitude from the tibia to the head showed that RaceRunning athletes were able to attenuate the impact shock throughout the stance phase of the running cycle. It was therefore suggested that the ergonomic design of the bike may serve as a mean to practice safe physical activity in terms of prevention of shock absorption-related injuries in people who are unable to walk unaided. Peters et al. [73] used a pressure mat between the athlete’s buttocks and the wheelchair seat to investigate the effect of wheelchair design parameters and the athlete’s physiological parameters on a peak pressure index and peak pressure gradient, since these parameters have formerly been identified as risk factors of developing pressure ulcers. The researchers found that lower pressure parameters correlated with higher seat angle and backrest, the type of cushion categorized by the authors as “therapeutic cushion” and a higher athlete BMI.

#### 3.5.3. Performance Characterization for Training Optimization

Several of the analyzed studies dealt with the usage of wearable sensors for sport performance characterization to optimize training [36,39,43,44,52,53,54,56,57,58,62,64,65,66,69,70,74], with EMG or inertial sensors being in some cases used in conjunction with other measurement systems (e.g., stereophotogrammetry and dynamometry). Wheelchair propulsion was investigated through the integrated analysis of mechanical data and EMG signals. For instance, in the study by Chow and colleagues [54], to guide teachers and trainers in choosing the more appropriate technique for wheelchair racing athletes, two racing wheelchair propulsion techniques (e.g., conventional and para-backhand) were compared in terms of kinematics and muscular activity of the upper limb. Minor differences were found in EMG signals from shoulder, arm and forearm muscles due to the large variation in muscle activation patterns within each technique group, while main difference between techniques was found in kinematic parameters. Even if not discriminative of an ideal propulsion technique, knowledge of muscular activation patterns relative to the different stages of a propulsive movement remains useful for coaches. Propulsion was also analyzed for handcycling movement by Faupin et al. [70] using EMG sensors, stereophotogrammetry and dynamometry, providing a case-study description of the movement phases. The analysis of muscle activation patterns and force output was also used to compare cross country sit-skiing propulsion between natural and simulated conditions (i.e., on snow versus ergometer) in Paralympic athletes [56]. According to similar muscle activation patterns and greater force output over time found in the simulated compared to natural condition, the cross country-ergometer was suggested to be a valid training tool for sport-specific maximal strength training and to test aerobic and anaerobic capacity. However, as declared by the authors, the lack of available trunk and upper limbs kinematic data limited the interpretation of the results, as only speculative explanation about the observed differences was possible.

Wheelchair mobility was also investigated adopting inertial sensors for performance description and analysis [41,42,43,44,45,64,66]. In Bergamini et al. [64], wheelchair propulsion performance in a 20-m sprinting test was evaluated in a team of junior wheelchair basketball players to identify the most adequate biomechanical performance indicators to develop discipline- and population-specific training programs by using inertial sensors. Sensor units were positioned on the wheelchair frame and on the wrists and were used to obtain parameters of propulsion timing, force and coordination. These parameters were then analyzed to enrich the design of a training program which, after being administered for 12 weeks, showed a better efficacy compared to classic training. In the study by van der Slikke et al. [41], inertial sensors positioned on the wheelchair frame were used to obtain 22 different kinematic outcomes, both linear and rotational, during wheelchair basketball gameplay at different competition levels. Authors identified a set of six parameters to quantify the wheelchair mobility performance in a standardized fashion, suggesting their implementation into the assessment process of athletes individual level. This set of parameters was then used in a subsequent work by the same research group [42] and by Haydon et al. in [45]. Mobility was also investigated in bicycle riding in a study by Cain et al. [65]. Inertial sensors were adopted to investigate the learning process of this motor skill in children with diverse disabilities. The bicycle was instrumented with two inertial sensors mounted on the frame and steering: it was found that, as the participants improved in motor performance, the correlation between angular velocity of frame and steering increased.

Swimming performance characterization was also carried out using inertial sensors [37,38,39]. Sensor units were positioned on Paralympic athletes’ body to investigate the role of kicking in freestyle swimming, by quantifying the kick count, rate and amplitude. In addition, in [39], the resulting force acting on the swimmer was measured using a custom-made dynamometer.

Monitoring of training load and its effect on sport performance was also investigated for training optimization [53,57,58,69]. EMG sensors were used to evaluate the effect of training load on muscular activity between different exercise intensities in sports such as Paralympic bench pressing [69], wheelchair racing [53,67] and handcycling [57,58]. In these studies, parameters of interest were muscle activation characteristics (onset, offset, range of activation) and indicators of muscular effort (integrated EMG signal). For instance, in the studies by Quittmann et al. [57,58], the change in muscle activity patterns during handcycling at continuous and increasing load was investigated in non-disabled participants. Results from these studies showed muscle-specific alteration in activation patterns due to the increasing muscular effort during the tests, thereby suggesting that specific muscle functions should be considered when designing training protocols. Training load was also monitored in-field by means of inertial sensors in wheelchair sports [36,49,50]. To investigate the changes in training load according to the specific player’s sport activity, sensor units were attached on the wheelchair frame to obtain parameters such as mean linear acceleration, rotational velocity and acceleration in wheelchair basketball [49] and wheelchair tennis [50] or on the participant’s body for computation of energy expenditure and intensity level (e.g., metabolic equivalent of the task) in wheelchair rugby [36]. Furthermore, heart rate sensors were used to monitor training load in running [61,62] and wheelchair basketball [49].

The research purpose of some studies dealing with sport performance characterization was to methodologically assess the validity and reliability of biomechanical systems based on wearable sensors or to compare data processing techniques [37,40,46,48,57,59,63], with some of them considering different gold-standard instruments to this purpose. In this regard, Mason et al. [40] and Laschowsky et al. [48] compared IMUs with camera-based laboratory systems, whereas Fulton et al. [37] proposed an in-field comparison of an inertial sensor-based system with an underwater camera. In Vieira et al. [46], a biomechanical model for the estimation of knee angle in indoor rowing based on the seat position, subject specific anthropometric measurement and two IMUs positioned on the lower limb was validated in non-disabled and Paralympic rowers. In the study by Laschowski et al. [48], a biomechanical model of Paralympic wheelchair curling was developed and validated with a single Paralympic athlete who was equipped with a 17-IMUs suit and digital goniometers. The model was used to measure angular velocities and compute joint moments using inverse dynamics during the wheelchair curling delivery. In another study, Sindall and colleagues [63] tested the accuracy and validity of two systems to track distance and speed in athletes playing wheelchair tennis: *(a)* a position data logger attached to the rear wheel axle, and *(b)* a GPS with embedded accelerometer. However, both systems were shown to underestimate distance and speed, calling for further development. In their study, Quittmann and colleagues [59] evaluated sport-specific maximal voluntary isometric contraction (MVIC) performed at different crank angles as a method to normalize EMG signal in handcycling in comparison to MVIC against manual resistance in non-disabled athletes.

Wearable sensors were also used to explore the effect of disability on physiological components of performance, with a particular interest in cerebral palsy (CP). EMG and heart rate sensors were adopted to investigate the neuromuscular and physiological characteristics of exercise-induced fatigue in Paralympic running athletes with CP compared to non-disabled ones [60,61,62]. In their first descriptive study, Runciman and colleagues [60] explored the effects of fatigue on muscle activity and power output during a maximal cycling test by analyzing the EMG signal in both frequency and amplitude domains and computing a fatigue index. In another study, the effect of induced volitional fatigue on sprint and jump performance was investigated in terms of performance-related outcomes (e.g., sprinting time and jumping height) and neuromuscular activity, with a particular focus on the symmetry between the affected and non-affected side in the athletes with CP [62]. In the third study, pacing strategy as a means to manage exercise-induced fatigue was tested through deceptive trials during shuttle running sets [61]. Results from these studies showed that athletes with CP do not present the fatigue resistance that is typical of untrained individuals with CP, likely due to the high training volumes that enabled them to produce performance characteristics similar to those of non-disabled athletes. However, authors concluded that a residual effect of CP on the body cannot be eradicated, as neuromuscular deficits can still be found in athletes with CP altogether with a conservative pacing strategy. Nevertheless, these findings might have important implication for athletic participation as a rehabilitation tool.

The effect of disability on physiological components of performance was also studied in relation to the motor adaptations to prosthetic cycling in people with trans-tibial amputation using EMG sensors altogether with stereophotogrammetry and dynamometry [52]. A modification in motor control was observed (e.g., delayed muscle recruitment onset), with specific muscle functions changing in order to control the prosthetic socket and reduce stress on residuum tissues at residuum-socket interface.

#### 3.5.4. Sports Equipment Customization

In five of the included studies, wearable sensors were used to assess the influence of sports equipment setup on performance and, subsequently, to provide information for setup optimization [42,45,47,70,73]. The most common type of investigated equipment was the wheelchair and its specific design parameters, including seat height [42,45,47], horizontal position or depth of the seat [45,47], seat angle [45,73], tire pressure [45], wheelchair mass [42], grip size/friction [42] and back rest height [73]. The effect of each wheelchair setting on wheelchair mobility performance was investigated with different research designs across the analyzed studies. For instance, in Haydon et al. [45] and in van der Slikke et al. [42] the individual “current setup” was used as baseline and then setup parameters were modified to test different wheelchair configurations, whereas in Masse et al. [47] the same standardized wheelchair configurations were adopted for all participants. In Peters et al. [73], a cross-sectional approach was used, with athletes being tested using their usual wheelchair setup. In these studies, the following performance-related parameters were obtained through the use of EMG sensors, IMUs or pressure mat: wheelchair linear and rotational acceleration and speed [42,45], muscle activation profiles of arm and shoulder [47] or pressure between the seat and the buttocks [73]. Results from these studies showed that seat height [42,45,47], seat depth [45,47] and seat angle [45] significantly affected wheelchair mobility of the athlete by changing the hand position at the time of rear wheel contact and release [45]. In addition, Haydon et al. [45] indicated that the tire pressure affected wheelchair mobility performance likely by changing the friction of the wheel with the ground. In the study by Masse et al., lower seat height and greater seat depth (seat further back) were associated with lower EMG activity of arm and shoulder muscles and smoother motion of upper limb joints, likely reducing the energy required for wheelchair propulsion. An increased wheelchair mass was shown to negatively affect the wheelchair mobility, while grip did not have any significant influence [42]. In Peters et al. [73], a greater seat angle and backrest height correlated with lower peak pressure between the athlete’s buttock and seat, representing a protective factor against the development of pressure ulcers. The effect of sports equipment setup on performance was also explored in handcycling, although it was not the primary aim of the study [70]. In their work, Faupin et al. [70] performed a biomechanical analysis of handcycling and, based on their results, suggested that parameters related to handbike configuration, such as crank adjustment, backrest angle and crank-backrest distance, may influence the handcycling performance in terms of muscular activity and kinematics of the upper limb.

## 4. Discussion

### 4.1. General Trends and Flaws

The aim of this review was to systematically evaluate current literature regarding the use of wearable sensors in sport for people with disabilities in different application fields. The growing interest in sport for people with disabilities has been mirrored by an increase in wearable sensor adoption in research production over the last 5-years period, as more than the half of the retrieved papers were published in this time window. Overall, many different types of wearable sensors were adopted to assess sport performance characteristics in 14 sport disciplines, with sensor configuration, disability and setting changing across the included studies according to the specific purposes. From a research quality perspective, the most common flaws of the included papers were the inadequate number of participants and description of strategy for subject selection. While the first issue is often due to a lack of power analysis report and likely related to the difficulty in recruiting and testing athletes with disability, especially Paralympians, the second issue is mainly due to meaningful information that are not always declared by the authors. In fact, not providing information such as comprehensive inclusion/exclusion criteria description allows a certain degree of uncertainty and variability related to subject selection into the analysis and interpretation of the results, thereby limiting the quality of findings.

### 4.2. Sensor Types and Placements

The most commonly used wearable sensors were inertial and EMG sensors, often in conjunction with other types of sensors (e.g., force sensor, GPS, digital goniometer or heart rate sensor). This is in line with what already observed in similar sports biomechanics literature involving non-disabled athletes [12,13], likely because they allow measuring the biomechanical and physiological characteristics of performance in a more ecological setting. Furthermore, EMG or inertial sensors were frequently used in combination with video analysis and motion capture systems to take advantage of both measurement systems strengths, allowing to combine measurements of muscle activity or body/equipment motion with joint kinematics [43,44,45,47,48,49,50,52,53,54,58,67,70,74]. It is worth noting that none of the analyzed papers adopted flexible, skin-interfaced wearable devices to monitor the physiological and biochemical status of the athlete with disability. Even though these sensors are predominantly adopted in healthcare monitoring, recent advances in this technology also offer the opportunity to continuously observe the changes in athletic parameters which are relevant to sports performance analysis or injury prevention [75,76]. Flexible wearable sensors could represent a true added value to continuously monitor health status together with the quality/quantity of physical exercise, which is of great importance for athletes with disability.

A crucial aspect that limits the transversal interpretability of results is the lack in standardization in positioning. In wheelchair sports, as in other sports analyzed with inertial sensors, sensor positioning protocols is a prerogative of each research group. One reason for this is likely the limited number of studies performed on the same sport. In the study of wheelchair propulsion, gyroscopes mounted on the wheelchair wheel axle were used to measure the angular velocity of the rear wheels, to estimate the speed of the wheelchair and to compute rotational and turning speed due to the differential steering of a wheelchair [41,42,45,50]. Instead, a different sensor configuration was used by Mason et al. [50] and Usma-Alvarez et al. [66] that mounted IMUs on the middle of the wheelchair and on the pick bar (bar positioned in front of the footplate which protects against tackles), respectively, to measure the wheelchair rotational acceleration. To overcome the limited comparability of results across different studies due to lack in standardized protocols, future studies should take into consideration previously published work when designing experimental protocols to facilitate building common knowledge.

In the studies adopting EMG sensors, motion tracking devices were fundamental for muscle activation pattern recognition and contextualization, providing kinematic information to obtain temporal events for the segmentation and analysis of the registered EMG signal. Specifically, video analysis [47,53,54,74], stereophotogrammetric systems [52,70] and other motion capture techniques [58,67] were used. Positioning of EMG sensors varied and was influenced by the observed sport activity and the specific purpose of the study, although particular attention was given to the investigation of trunk and upper limb muscles, which played a predominant role in determining performance as the majority of the reported disabilities affected the lower body.

Interestingly, none of the analyzed papers used EMG and inertial sensors in combination, as similarly observed in non-disabled athletes [13]. This integration has a wide potential from two different perspectives, since IMUs can be used to: (a) estimate resultant forces from acceleration [64]; (b) accurately estimate three-dimensional orientation of body segments and compute joint kinematics (see also Section 1.2 and the study by Vieira and colleagues [46]). Both pieces of information are relevant and, if monitored along with the EMG signal, could provide a comprehensive picture not only of the biomechanics of a specific motor task, but also of the underlying physiology even in in-field condition.

In few studies, force sensors were used to provide kinetic parameters in conjunction with both EMG [56,70,71] or inertial sensors [39]. When combined with muscular activity measurements, kinetic data was used to evaluate the athlete’s maximal capacity of force exertion and muscle activation in a newly proposed field test [71], or to obtain force output profiles in relation to muscle activation patterns during cyclic activities, such as handcycling [70] and cross-country sit skiing [56]. Force output was also related to sport-specific kinematic parameters, such as the kick rate during swimming [39], in combination with inertial sensors. The evaluation of the forces that act upon or are generated by the athlete is critical for performance analysis and injury prevention; literature, however, seems still at its pioneering stage in this respect.

Therefore, there is evidence that different combination of wearable sensors should be used to provide exhaustive and valuable information about sport performance increasing the possibility of obtaining biomechanical measurements in unusual outdoor conditions. Furthermore, the crucial role of kinetic quantities in performance analysis and injury prevention, especially in ecological conditions, calls for further research to overcome technical limitations allowing to explore the kinetic perspective.

### 4.3. Parameters and Applications

Many of the applications of wearable sensors in sport biomechanics of non-disabled athletes that were indicated by previous reviews (i.e., injury prevention, performance characterization for training optimization) are applied to athletes with disabilities at a growing rate. In addition, new contexts of wearable technology application specific to sport for people with disability are reported, such as athlete classification and sports equipment customization. Parameters and methods for all the above-mentioned applications are discussed in detail in the following subsections.

#### 4.3.1. Athlete Classification

Technology can provide the ideal tools for a more objective classification process for athletes with disability by assessing how sport performance is affected by the functional limitations descending from the impairment [28]. Only few studies were specifically performed to measure the effect of impairment on the athlete’s functional ability investigating features specific to the sport discipline [55,68,71], for example trunk strength in cross-country sit-skiing [68]. EMG, inertial and force sensors were used to measure maximal capacity of force exertion and muscle activation [68,71], trunk segment range of motion [68] and electromechanical delay between onset of muscle activation and motor task execution [55]. These studies were not able to provide adequate athlete classification measurement systems, either because they did not show enough sensitivity to discriminate different sport classes [55] or because of limited sample size [68]. Nevertheless, these studies indicated the potential of the measured parameters for athlete classification that should be considered for future research on this topic. Furthermore, a common denominator across the papers dealing with athlete classification was the evaluation of the role of trunk in sport performance [68,71], as it plays an important role in force production and transmission during various propulsion-related sport activities, from wheelchair propulsion to sit-skiing. In fact, trunk motor behavior can be easily assessed through wearable inertial sensors to extract kinematics [48] and EMG sensors to evaluate the muscle activity related to trunk stabilization [71]. However, for most Paralympic sports, further studies with greater sample size involving athletes with different classification are still needed to provide validated and reliable tests for more objective procedures for athlete classification.

#### 4.3.2. Injury Prevention

The application of wearable sensors in the analysis of sport technique aimed at injury prevention has been previously reported [5,30]. Specific applications for people with disability were quite scarce. Furthermore, the three retrieved studies differed for sports, sensors, injury types and study designs. Nevertheless, these papers were in line with recent investigation of the frequency, types and causes of injury in adaptive summer sports, such as wheelchair basketball, wheelchair rugby, swimming and athletics [6]. In particular, pressure ulcer along with bone fracture and overheat illness were the most common injuries in wheelchair sports. Pressure mat was used to measure pressure-related parameters that were found to be associated with both wheelchair and athletes characteristics in the study by Peters et al. [73]. Of no second importance are rotator cuff injuries, which were targeted by Barfield and colleagues [51] through analysis of EMG signals in wheelchair rugby players, since they were reported to be by far the most common cause of long-term retirement from sport activity (over one year of absence from sport) in this population. Lower limbs injuries, mostly caused by overuse, were the most common in running. This aspect was investigated through the use of inertial sensors by Shafizadeh et al. [72], who analyzed strategies for impact shock absorption in athletes performing RaceRunning, an adapted version of on-track running.

Similar types of injury can be found spanning across different sport disciplines, such as shoulder injury in wheelchair sport and in swimming. Therefore, athletes with different sport-related needs could transversely benefit from wearable systems that are able to monitor the kinematics and/or muscle activity of the shoulder, such as the one adopted in Barfield et al. [51]. In addition, other types of transversal injuries across sports like heat illness are caused by changes in physiological parameters (e.g., body temperature) that can be easily monitored through wearable sensors. However, extensive research on injury prevention through the use of wearable sensors in sport for people with disability is still missing. Interestingly, a similar call for studies adopting wearables for injury prediction was also made in the context of sport for non-disabled people [12]. In their review, Adesida and colleagues highlighted the lack of identification of sport-specific biomechanical parameters that are obtainable from wearable sensors and are able to predict injury. In the context of sports for persons with disability, determining these parameters is more complex, as it does not only involve sport-specific aspects, but also includes the type and severity of disability [77]. For all athletes, therefore, particular attention should be focused on the definition of these parameters, resolving the complexity of predictors of injury also taking advantage of more recent data science techniques, with artificial intelligence representing a promising tool to address this question [78].

#### 4.3.3. Performance Characterization for Training Optimization

The most common application for wearable sensors in sport for people with disability was technique analysis for performance characterization, a trend that has been also reported in previous works on athletes without disability [5,12,13]. In sport for people with disability, studies explored classic aspects of sport biomechanics in non-disabled athletes, such as analysis of technique/performance [36,39,43,44,54,56,64,65,66,70,74], training load [53,57,58,69], biomechanical measurement system validation [37,40,41,46,48,63] and comparison of data processing techniques [59], but also components more specific to the observed population, that is, for example, how a specific disability affects motor and sport performance [52,60,61,62].

The effectiveness and appropriateness of current training practices in sport for people with disability have been questioned as there is a lack in research on sport-specific performance and development of athletes [79]. In the revised studies, wearable technologies were adopted to assess sport performance in a variety of different sports, with wheelchair propulsion and mobility tasks being the sport-related motor tasks of greatest interest. Inertial and heart rate sensors were used to monitor the overall physical activity during gameplays for the optimization of physical and technical training strategies in wheelchair court sports [36,49,50]. This is in line with Paulson et al. [80], who reported that the amount of physical activity was regarded as an important parameter in wheelchair court sports for both people with and without disability in order to prescribe training load to yield optimal performance. Furthermore, inertial sensors were also used to obtain more specific kinematic parameters which are informative of the athlete’s wheelchair propulsion performance and technique [41,43,44,45,64,66]. EMG sensors were found to offer the opportunity of monitoring the muscle activation patterns which are peculiar of propulsive technique [54,67], but also the change in muscle activity during wheelchair propulsion at different loads [53]. The example of wheelchair sports was only one of the possible applications of wearable sensors for training monitoring and optimization in athletes with disability, as demonstrated by the studies on other sports disciplines such as handcycling [57,58,70], cross-country sit-skiing [56] and swimming [37,38,39]. However, there are still some gaps in the available knowledge on the use of wearables that regards the scarcity of studies on specific sport disciplines and the lack of common evidence-based practice in the adoption of sensors in both research and daily sport activity. Future works should therefore aim at the validation of wearable measurement systems and their implementation into the diverse sports daily practice in order to provide solid quantitative background to the choices that trainers and coaches made during the training design process.

An application of wearable sensors which was found to be specific to sport for people with disability was the evaluation of the effect of disability on the biomechanics and physiology of sport and motor performance. In four analyzed studies, the comparison between athletes with and without disability allowed for the identification of peculiar changes in motor performance which are implemented by the athletes to deal with the limitations imposed by the impairment [52,60,61,62]. In particular, wearable sensors such as EMG, force and heart rate sensors can be effectively adopted to assess muscle activity, power and fatigue when these aspects are of interest for the study of impairment, for example in the evaluation of fatigue management in athletes with CP [60,61,62]. Furthermore, although no inertial sensors were adopted in these studies, the adoption of stereophotogrammetric system in Childers et al. [52] would suggest the possible application of inertial sensors for the computation of joint kinematics while evaluating the motor adaptation of prosthetic cycling. Wearable technologies would therefore offer the opportunity to compare athletes with and without disability in real sport-life conditions. This would provide information about the effect of specific impairment on motor control which is essential not only for advancing in the medical knowledge, but also for sport and rehabilitation professionals in order to personalize training protocols based on the individual specific strategies.

#### 4.3.4. Sport Equipment Customization

In common practice, sports equipment is customized to the needs of each specific athlete with disability and this procedure is typically performed with the help of skilled and experienced coaches [10]. However, due to various reasons, not all athletes may have access to an experienced coach. As stated in Section 3.5.4, there is some evidence pointing towards the possibility to use wearable sensors as a support for the adjustment of sport equipment, especially to assess the effect of different design parameters on wheelchair mobility performance. In particular, seat height, seat depth, seat angle, tire pressure, and back rest height modulate wheelchair propulsion and, therefore, are of interest when customizing the equipment to the athlete’s needs [42,45,47,73]. In addition to wheelchair setup optimization, one study dealing with handcycling provided further insight on the use of wearable sensors in sport equipment customization. Faupin et al. [70] indicated that EMG activity and kinematics of the upper limb could be affected by crank adjustment, backrest angle and crank-backrest distance, thereby suggesting the additional use of EMG sensors to monitor the effects of various handbike design configuration on handcycling performance. The enormous opportunity for the development of technologies that are capable of monitoring the sport performance in relation to the selection of safe and performance-enhancing sports equipment was also indicated in a recent review by Cooper et al. [29]. The authors also proposed the use of wearable IMU-based actigraphs to assess energy expenditure over time during wheelchair sport as a parameter to evaluate wheelchair configurations. Current literature on the use of wearable sensors for wheelchair customization suggests that other sport disciplines, such as rowing or archery, could benefit from the same application of wearable sensors.

## 5. Conclusions

Wearable sensors provide a promising opportunity to quantitatively assess the individual functional capacities of the athlete with disability in an ecological environment. The available evidence for the application of wearable sensors in sport for athletes with disabilities is mainly focused towards performance assessment in wheelchair sports. Main performance indicators included linear and rotational wheelchair accelerations and the amount of upper body muscle activity measured by inertial and EMG sensors, respectively. The available scientific literature concerning applications specific to sports for people with disability, such as athlete classification and injury prevention, although limited, shows a possible direction for further development. Future approaches in dealing with athlete classification and injury prevention should consider the definition of biomechanical and physiological parameters relevant to the athletic performance on a sport-specific basis and investigate their association with the functional limitations related to the type and severity of disability. Applications of wearables application in performance characterization for training optimization mirrored classic aspects of sport biomechanics in non-disabled athletes, but also investigated the effect of disability on sport performance. Although underexplored, this field of application is of particular interest for the community of coaches, trainers and athletes with disability as it can provide useful information for all the other above-mentioned contexts of application. Furthermore, acquiring additional knowledge about the athletic performance will help in translating current evidence from sports for non-disabled people to adapted sports. Finally, since the equipment is frequently of particular importance in sports for persons with disability, literature indicates that wearable systems are promising to support the customization of equipment to meet the athlete individual needs.

## Figures and Tables

**Table 1:** Inclusion and exclusion criteria.

Criteria:	Definition:
Measurements	If wearable: Inertial measurement units Electromyographic sensors Force transducers Pressure sensors Other sensors measuring physiological signals (i.e., heart rate, oxygen consumption)
Motor tasks	Included: Sport-related movements Excluded: Everyday physical activity
Cohorts	Included: Persons with physical disabilities Non-disabled persons performing adaptive sport tasks Excluded: Persons with cognitive disabilities only
Type of assessment	Included: Quality of sports related movement Quantity of sports related movement Risk of injury Validation of technology or methodology implemented in sport for disabled people Excluded: Response to medical treatments and devices

**Table 2:** Number of included papers per each investigated sport.

Sports	
Wheelchair basketball	6
Wheelchair rugby	6
Wheelchair racing	5
Running	4
Handcycling	4
Swimming	3
Cross-country sit-ski	2
Wheelchair tennis	2
Cycling	2
Rowing	1
Paralimpic weightlifting	1
Wheelchair curling	1
Boccia	1
Downhill skiing	1

**Table 3:** Parameters measured with wearable sensors in sport for disabled people and the applications where the parameters have been used. AC = Athlete Classification; IP = Injury Prevention; PC = Performance Characterization; EC = Equipment Customization.

Sport-Related Movement	Sensor Type	Parameter	AC	IP	PC	EC	Reference
Wheelchair propulsion	Inertial sensors	Bilateral symmetry of acceleration Push cycle duration Progression force Push cycle frequency Cycle variation of parameters			√		[64]
		Peak linear acceleration			√		[43,44]
		Angular velocity of wheel			√		[40]
	EMG	Muscle activation pattern in shoulder and arm muscles				√	[47]
		Muscular activation pattern in back and abdominal muscles			√		[67]
		Mean EMG amplitude Peak EMG amplitude in arm muscles			√		[53]
		Mean EMG amplitude for different stroke phases and whole cycle in arm and back muscles			√		[54]
	Interface pressure mat	Pressure peak Pressure gradient		√		√	[73]
Wheelchair agility	Inertial sensors	22 kinematic outcomes related to linear and rotational speeds. Reduced to the 6 most important: (1) Mean of the five best rotational speeds in a turn; (2) Mean rotational acceleration; (3) Mean forward acceleration form first 2 m from standstill; (4) Mean forward speed; (5) Mean rotational speed in a curve; (6) Mean of five best forward speeds.			√	√	[41,42,45]
		Instantaneous turning radius Tangential velocity			√		[66]
Wheelchair rugby gameplay	Inertial sensors	Energy expenditure Intensity level Physical activity time			√		[36]
	EMG	Muscular activation pattern in deltoids and pectoralis		√			[51]
Wheelchair basketball gameplay	Inertial sensors	Wheelchair frame rotation and acceleration			√		[49]
Wheelchair tennis gameplay	Inertial sensors	Wheelchair mean acceleration rotational velocity and acceleration			√		[50]
	GPS with accelerometer	Speed Distance			√		[63]
Hand cycling	EMG	Integrated EMG			√		[57]
		EMG onset and offset EMG amplitude in upper body muscles			√		[57,58]
		Peak EMG amplitude in upper body muscles			√		[59]
		Percentage of muscular activation in arm and back muscles			√	√	[70]
Cycling	Inertial sensors	Peak cross-correlation between roll angular velocity and steering rate			√		[65]
	Force sensors	Pedal reaction force			√		[52]
	EMG	EMG onset and offset			√		
Wheelchair curling draw shot delivery	Inertial sensors	Angular displacement and velocity of shoulder, elbow wrist and hip			√		[48]
	Goniometers	Range of motion of shoulder, elbow wrist and hip			√		
Poling (Sit-ski)	Inertial sensors	Trunk range of motion	√				[68]
	Force sensors	Force production	√		√		[56,68]
	EMG	Peak EMG amplitude Mean EMG amplitude in upper limbs			√		[56]
Turning (Downhill skiing)	EMG	Muscular activation pattern in glute, thigh and leg			√		[74]
Benchpress	EMG	Percentage of muscle activation in upper body muscles			√		[69]
	Force sensors	Anterior force Posterior force Ratio between them	√				[68]
Running	EMG	Mean EMG amplitude in leg and lower back muscles			√		[60,61]
		Peak EMG amplitude in leg and lower back muscles			√		[62]
	Inertial sensors	Peak impact acceleration at tibia and head		√			[72]
Vertical jump	EMG	Mean EMG amplitude in leg and lower back muscles			√		[61]
Throwing a ball	EMG	Electromecanical delay	√				[55]
Swimming	Inertial sensors	Kick rate Kick amplitude			√		[37,38,39]
	Force sensors	Net force			√		[39]
Rowing	Inertial sensors	Knee angle			√		[46]
Upper body pushing force exertion (Wheelchair basketball)	Force sensors	Exerted force	√				[71]

## Supplementary Materials

The following is available online at https://www.mdpi.com/1424-8220/21/5/1858/s1, Supplementary Material Summary Table contains, for each included study: Author names, Title, DOI/ISSN, Publication year, Journal/Conference, Sport, Sport-related movement, Participants’ disabilities, Setting, Sample size, Groups of athletes, Athlete level, Aim/s of research, Intended benefit, Sensor types, Wearable sensor placement, Sampling frequency, Signal filter, Data transmission, Parameters (all), Parameters extracted with inertial sensors, Parameters extracted with EMG sensors, Parameters extracted with other types of sensors, Conclusions, Quality evaluation score. Legends for abbreviations for a given column are found hovering the cursor over the column heading.

## Abbreviations

The following abbreviations are used in this manuscript:

IMU	Inertial Measurement Unit
EMG	Electromyography
MVIC	Maximal Voluntary Isometric Contraction
MEMS	Microelectromechanical systems
IPC	International Paralympic Committee
CP	Cerebral Palsy

## Appendix A. Search Strings Used to Search in Databases

**Table A1:** Boolean search strategy strings for each database

Database Keywords
**Scopus:** TITLE-ABS-KEY ((wearab* OR acceleromet* OR gyro* OR *emg OR electromyo* OR *imu OR “inertial sensor” OR “inertial measurement unit*” OR mems OR “force sensor” OR “force transducer” OR “pressure sensor” OR (“energy expenditure” AND wearab*) OR (“heart rate” AND wearab*) OR (“oxygen consumption” AND wearab*) OR (vo2* AND wearab*))
AND (sport* OR “physical training” OR athlet* OR basketball OR fenc* OR rugby OR tennis OR curl* OR archery OR athletics OR badminton OR boccia OR canoe* OR bik* OR cycling OR cyclist OR equestrian OR football OR soccer OR judo OR “weight lift*” OR “power lift*” OR powerlift* OR row* OR shoot* OR swim* OR “table tennis” OR biathlon OR triathlon OR volleyball OR ski OR skiing OR hockey OR snowboard* OR taekwondo OR “martial art” OR sail* OR “track and field” OR fishing OR golf OR hiking OR hunting OR hunter OR kayak* OR paddl* OR raft* OR climb* OR scuba OR diving OR diver OR dive OR skateboard* OR snowshoe* OR “strength train*” OR surfing OR surfer OR “tai chi” OR racing OR race OR yoga OR “adaptive sport” OR running OR runner OR jog*)
AND (disab* OR paraly* OR prosth* OR handi* OR impairment OR impaired OR amput*))
**Web-of-Science:** TS=((wearab* OR acceleromet* OR gyro* OR *emg OR electromyo* OR *imu OR “inertial sensor” OR “inertial measurement unit*” OR mems OR “force sensor” OR “force transducer” OR “pressure sensor” OR (“energy expenditure” AND wearab*) OR (“heart rate” AND wearab*) OR (“oxygen consumption” AND wearab*) OR (vo2* AND wearab*))
AND (sport* OR “physical training” OR athlet* OR basketball OR fenc* OR rugby OR tennis OR curl* OR archery OR athletics OR badminton OR boccia OR canoe* OR bik* OR cycling OR cyclist OR equestrian OR football OR soccer OR judo OR “weight lift*” OR “power lift*” OR powerlift* OR row* OR shoot* OR swim* OR “table tennis” OR biathlon OR triathlon OR volleyball OR ski OR skiing OR hockey OR snowboard* OR taekwondo OR “martial art” OR sail* OR “track and field” OR fishing OR golf OR hiking OR hunting OR hunter OR kayak* OR paddl* OR raft* OR climb* OR scuba OR diving OR diver OR dive OR skateboard* OR snowshoe* OR “strength train*” OR surfing OR surfer OR “tai chi” OR racing OR race OR yoga OR “adaptive sport” OR running OR runner OR jog*)
AND (disab* OR paraly* OR prosth* OR handi* OR impairment OR impaired OR amput*))
**EBSCO:** Same as Web-of-Science
**Pubmed:** ((“Electromyography”[Mesh] OR “Accelerometry”[Mesh] OR “Wearable Electronic Devices”[Mesh]) AND “Sports for Persons with Disabilities”[Mesh])
OR (wearab* OR acceleromet* OR gyro* OR *emg OR electromyo* OR *imu OR “inertial sensor” OR “inertial measurement unit*” OR mems OR “force sensor” OR “force transducer” OR “pressure sensor” OR (“energy expenditure” AND wearab* ) OR (“heart rate” AND wearab* ) OR (“oxygen consumption” AND wearab* ) OR ( vo2* AND wearab* ) )
AND (sport* OR “physical training” OR athlet* OR basketball OR fenc* OR rugby OR tennis OR curl* OR archery OR athletics OR badminton OR boccia OR canoe* OR bik* OR cycling OR cyclist OR equestrian OR football OR soccer OR judo OR “weight lift*” OR “power lift*” OR powerlift* OR row* OR shoot* OR swim* OR “table tennis” OR biathlon OR triathlon OR volleyball OR ski OR skiing OR hockey OR snowboard* OR taekwondo OR “martial art” OR sail* OR “track and field” OR fishing OR golf OR hiking OR hunting OR hunter OR kayak* OR paddl* OR raft* OR climb* OR scuba OR diving OR diver OR dive OR skateboard* OR snowshoe* OR “strength train*” OR surfing OR surfer OR “tai chi” OR racing OR race OR yoga OR “adaptive sport” OR running OR runner OR jog*)
AND (disab* OR paraly* OR prosth* OR handi* OR impairment OR impaired OR amput*))
